# Social Mindfulness Shown by Individuals With Higher Status Is More Pronounced in Our Brain: ERP Evidence

**DOI:** 10.3389/fnins.2019.01432

**Published:** 2020-01-20

**Authors:** Juanzhi Lu, Xiaoxuan Huang, Chong Liao, Qing Guan, Xin-Rui Qi, Fang Cui

**Affiliations:** ^1^School of Psychology, Shenzhen University, Shenzhen, China; ^2^Center for Translational Neurodegeneration and Regenerative Therapy, Shanghai Tenth People’s Hospital Affiliated to Tongji University School of Medicine, Shanghai, China; ^3^Shenzhen Key Laboratory of Affective and Social Cognitive Science, Shenzhen University, Shenzhen, China; ^4^Center for Brain Disorders and Cognitive Neuroscience, Shenzhen, China

**Keywords:** social mindfulness, low-cost cooperation, social status, ERP, FRN

## Abstract

“Social mindfulness” refers to being thoughtful of others and considering their needs before making decisions, and can be characterized by low-cost and subtle gestures. The present study compared the behavioral and neural responses triggered by observing others’ socially mindful/unmindful choices and how these responses were modulated by the social status of the agency. At the behavioral level, observing socially mindful choices made observers feel better, rate the actors as more likable, and behave more cooperatively than did observing socially unmindful choices. Analysis of event-related potentials in the brain revealed that compared with socially unmindful choices, mindful choices elicited more negative feedback-related negativity (FRN). Notably, while this effect of social mindfulness was only significant when the actor’s social status was medium and high, it was undetectable when the actor’s social status was low. These results demonstrate that the social mindfulness of others can be rapidly detected and processed, as reflected by FRN, even though it does not seem to receive further, more elaborate evaluation. These findings indicated that low-cost cooperative behaviors such as social mindfulness can also be detected and appreciated by our brain, which may result in better mood and more cooperative behaviors in the perceivers. Besides, the perception of social mindfulness is sensitive to important social information, such as social status.

## Introduction

Imagine you are in line to buy a pie and there are two strawberry pies and one cherry pie remaining. If the person in front of you takes a strawberry pie instead of the last cherry pie, you might feel this was a kindness because it left you with both options. “*Social mindfulness*” refers to being thoughtful of others and considering their needs before making decisions (Van Doesum et al., 2013; Van Lange and Van Doesum, 2015). For people waiting in line with others behind them, if they choose the last cherry pie, the next people in line will no longer have the possibility to choose. This behavior can be seen as “socially unmindful.” In contrast, if they choose one of the strawberry pies, the next person in line will still have both options. This behavior is considered “socially mindful.” One aspect of being socially mindful is leaving more control over outcomes to others (Lemmers-Jansen et al., 2018), which is highly valued in society (Aoki et al., 2014).

**FIGURE 1:**
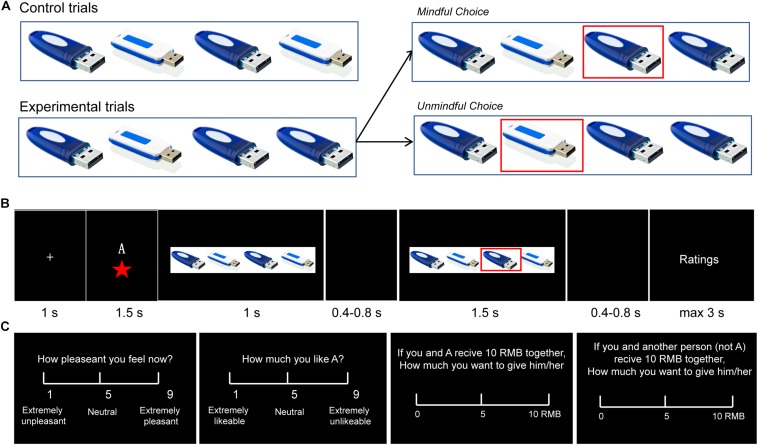
A experimental design. **(A)** Control trials (2:2 or 3:0 ratios) and experimental trials (3:1 or 2:1). In the experimental trials, if the participant chose the unique item, it was classified as “unmindful”; otherwise if the participant chose one of the other items, it was classified as “mindful.” **(B)** Trial structure. In each trial, after a 1 s fixation, the actor’s (A’s) social status was presented for 1.5 s, followed by the objects for 1 s. After a 0.4–0.8 s blank period, A’s choice was marked with a red square that lasted for 1.5 s. After another 0.4–0.8 s blank period, the participant was asked to questions about A’s choice. **(C)** The four questions were presented in different blocks. (1) “How pleasant do you feel now?” (Rating on a 1–9 scale: 1 = extremely unpleasant, 9 = extremely pleasant); (2) “How much do you like A?” (Rating: 1 = extremely likable, 9 = extremely dislikable); (3) If the experimenter gives you 10 RMB (Chinese currency) to share with A, how much would you give to A? (from 0 to 10 RMB) (Dictator Game; DG); and (4) If the experimenter gives you 10 RMB to share with some other person (not A) how much would you give to the person? (from 0 to 10 RMB) (DG with the other (not A).

The paradigms used in the literature of human cooperation usually involve explicit monetary cost. However, in real life, we cannot always explicitly perceive another’s need, and as in social mindfulness, substantial costs are not always needed to be cooperative (Klapwijk and Van Lange, 2009; Van Lange et al., 2012; Balliet and Van Lange, 2013). The Social Mindfulness (SoMi) task is designed to investigate this kind of low-cost cooperation. Participants are presented with a set of three or four items among which one is unique from the rest (e.g., three blue U-disks and one white U-disk, Figure 1A). They are asked to choose one of these items, and then another person chooses from the remaining ones: (1) if participants choose the unique item and leave the others with no choice, it is counted as socially unmindful, and (2) if they choose one of the non-unique items and leave the others with more options, it is counted as socially mindful (Van Doesum et al., 2013; Van Lange and Van Doesum, 2015). Though research regarding social mindfulness is still in its infancy, a few studies have already shown that it can be noticed and appreciated by observers on behavioral level, and lead to more positive social judgments, even further cooperation (Van Doesum et al., 2013; Dou et al., 2018b).

Social status is the relative rank of an individual along one or more dimensions within a given social hierarchy, which can strongly modulate human socio-emotional functioning and attention/cognitive processes (Zink et al., 2008; Boksem et al., 2012; Santamaria-Garcia et al., 2014; Feng et al., 2015, 2016). More importantly, social status is a factor that influences how we evaluate the behavior of others (Mattan et al., 2017; Ren et al., 2018; Rizzo and Killen, 2018). Previous studies have found that social status is an influencing factor in how we behave and how we perceive other’s behavior in cooperation games. For instance, a study has reported that in Ultimatum Game (UG), high-status participants reject more unfair offers than do low-status individuals (Hu et al., 2014, 2016). Another study found that when participants played cooperative games with others who had different levels of social status, they showed a significant subsequent decrease in cortisol concentration only when they were paired with a higher status co-player (Michael et al., 2016).

During interpersonal interactions, people consistently perceive and evaluate signals from others. These signals can be explicit (e.g., an unfair offer proposed directly) or implicit (e.g., a rude, unthoughtful gesture). How we perceive these signals from others dictate how we react. For example, a study reported that the feedback-related negativity (FRN) elicited when participants observe others making unfair offers in an UG can predict the participant’s decision to reject the offers (Hewig et al., 2011). FRN is a negative event-related potential (ERP) component that occurs at the frontocentral region, peaking roughly 250–300 ms after the presentation of feedback information (Gehring and Willoughby, 2002; Cohen et al., 2007). It has been consistently found to be more negative for a negative outcome than for a positive outcome (e.g., FRN is usually larger for monetary losses than for gains) (Gu et al., 2010, 2011). Recent studies have suggested that FRN is elicited by unexpected outcomes regardless of valence and that it actually indicates the absolute difference between the actual and expected outcome (Oliveira et al., 2007; Ferdinand et al., 2012). The motivational theory of FRN has been proposed, which states that outcomes that are more motivationally significant lead to enhanced FRN (Zhou et al., 2010; Ma et al., 2015). Evidence supporting the motivational theory of FRN comes from studies showing that FRN elicited by highly self-relevant outcomes is larger than that elicited by low self-relevant outcomes (Li et al., 2010; Ma et al., 2011). In general, FRN reflects a rapid and coarse evaluation of the perceived feedback signal. It is sensitive to feedback signals in non-social contexts (e.g., monetary gains or losses) as well as those in social contexts [e.g., social norm violation (Mu et al., 2015)]. Positive-going P3 is an ERP component that follows the FRN, and which is most pronounced at parietal electrode sites around 300–500 ms after feedback onset (Polich, 2007; Kobza et al., 2011). P3 has been reported to be modulated by multiple contextual social factors and is considered to reflect a more elaborate and cognitive evaluation of the feedback (Chen et al., 2017).

The present study aimed to explore how socially mindful and unmindful behaviors are perceived differently by observers both behaviorally and in the brain. Considering that social status plays a crucial role in costly cooperation scenarios, another aim of the present study was to explore how social status may play a part in the processing of social mindfulness. Here, we recorded electroencephalograms (EEG) while participants observed pre-collected data depicting another group of participants with different social statuses doing the SoMi task. We asked participants to rate the actors as they observed mindful and unmindful choices. Behavior and EEG data were then compared systematically.

At the behavioral level, we hypothesize that compared with observing socially unmindful choices (i.e., choosing the unique item), observing socially mindful choices (i.e., choosing the non-unique item) would induce a more pleasant mood, the actor would be rated as more likable, and the participants would behave more cooperatively. At the neural level, since the other’s behavior in the SoMi task reflects how thoughtful and cooperative the actor is, to some extent, it can be considered as an implicit social signal that the observers would perceive, evaluate, and adjust their own behavior to accordingly. Thus, we predicted that the FRN and/or P3, which reflect general evaluation processes, would be sensitive to the social mindfulness of others. Additionally, previous studies suggest more attention would be allocated to process information regarding individuals with higher social status (Mattan et al., 2017). We, therefore, predicted that the effect of social mindfulness would be enhanced when the perceived actor’s social status increased.

## Materials and Methods

### Ethics Statement

All research procedures were approved by the Medical Ethical Committee of the Medical School in Shenzhen University according to the Declaration of Helsinki. All participants gave their written informed consent after they fully understood the study’s instructions.

### Participants

Thirty right-handed participants at Shenzhen University participated in the study. Data from two participants were rejected due to intensive head movements during the EEG recording (rejected over 25% trials). Thus, 28 participants were included in the final data analysis (14 females, 19.54 ± 1.91 y). Participants were screened for a history of neurological disorders, brain injury, and developmental disabilities. All had a normal or corrected-to-normal vision. The sample size was calculated using a prior power analysis in G^∗^Power 3.1.7. 24 participants were needed to reach a power of 0.9.

### Design and Procedures

#### Establishing Social Status

To establish the social hierarchy, all participants were asked to perform a Code Input task in which participants were given 3 minutes to input codes. Each code was composed of five random numbers and letters. The participants were informed that their performance in this task would be evaluated by the numbers of correctly input codes. Then they would be ranked as low (one star), medium (two stars), or high (three stars) status accordingly. Unknown to the participants, the outcome of the task was manipulated such that all participants were ranked as a medium. A similar procedure has been employed in previous studies, and participants have been demonstrated to be strongly engaged in this social hierarchical context (Zink et al., 2008; Breton et al., 2014; Santamaria-Garcia et al., 2014; Feng et al., 2016).

#### The Self-SoMi Index

After completing the Code Input task to establish their own status, the participants performed the standard SoMi task. They were told that they would perform a task with another participant who they had never met and would not likely knowingly meet again in the future. In actuality, the other “participant” was a confederate who won’t show in person. Participants were presented with pictures showing three or four items that were either completely identical or only slightly different (e.g., different in color). There were two conditions: In experimental trials with three or four items, one of the items always differed from the others (one unique vs. two [1:2] or one unique vs. three identical [1:3] items). In control trials, no items were unique (three identical [0:3] or two identical vs. two identical [2:2] items). The participants were told that they were to take turns picking items. The real participant was told to pick first. Note, participants were also informed that the items would not be replaced once chosen and that they would not be available to the other participant. The SoMi task included 24 trials (12 experimental and 12 control) and lasted about 10 min. Each participant’s SoMi index was then calculated as the proportion of socially mindful decisions, varying from 0 (only social unmindful choices) to 1 (only socially mindful choices) (Lemmers-Jansen et al., 2018).

#### ERP Experiment

The participants were then seated comfortably in the EEG room. They were informed that they would now be shown how other participants (termed “actors” in the following text) performed on the SoMi task in a previous study. Each trial presented the choice of a different actor. In each trial, after a 1 s fixation, participants were shown the actor’s social status (i.e., one, two or three-stars player) for 1.5 s. Then participants were shown the array of choices (three or four items) for 1 s, which indicated the type of trial (control: 2:2 or 3:0 item ratio; experimental: 3:1 or 2:1). After a 0.4–0.8 s blank screen, the actor’s choice (mindful or unmindful in the experimental trials) was marked for 1.5 s with a red frame around the selected item. After that, participants were instructed to answer four questions (see below) on a scale of 1 to 9 (Figures 1A,B). Participants had 3 s to respond to each question. The ERP experiment contains 4 blocks of 81 trials (324 trials in total) and lasted about 45 min. In each of the four blocks, there were 27 control trials and 54 experimental trials that depicted mindful or unmindful choices. The order of the trials was pseudo-randomized.

One of the following four questions was presented after each trial in a given block. (1) “How pleasant do you feel now?” (1 = extremely unpleasant, 9 = extremely pleasant); (2) “How much do you like A?” (“A” was the actor) (1 = extremely likable, 9 = extremely dislikable); (3) “If the experimenter gives you 10 RMB to share with A, how much would you give to A?” (from 0 to 10 RMB) (Dictator Game; DG); and (4) “If the experimenter gives you 10 RMB to share with some other person (not A) how much would you give to the person?” (from 0 to 10 RMB) (DG with the other (not A) (Figure 1C). The first two questions were designed to assess how observing other’s social mindfulness modulates an observer’s own mood and their feelings toward the actors. The latter two questions were designed to assess how observing other’s social mindfulness influences an observer’s future decision-making.

### EEG Acquisition and Analysis

Electroencephalography (EEG) data were recorded from a 64-electrodes scalp cap using the 10–20 system with a sampling frequency of 1000 Hz (Brain Products, Munich, Germany). The electrode at the right mastoid was used as the reference during recording while the electrode on the medial-frontal aspect was used as a ground electrode. Two electrodes were used to measure the electrooculogram (EOG). EEG and EOG activity was amplified at 0.01 Hz–100 Hz band-passes. All electrode impedances were maintained below 5 kΩ. EEG data were pre-processed and analyzed using BrainVision Analyzer 2.0.1 (Brain Products GmbH, Germany). EEG data were re-referenced to a common average. Then the signal passed with 0.01–30 Hz band-pass filter. Possible artifacts (eye movements and blinks, cardiac signals, and line noise) were corrected using an independent component analysis (ICA) (Jung et al., 2001). Segmented EEG data were stimulus-locked to the onset of the outcome of the offer. The ERP epochs were trimmed (from −200 ms to 800 ms) and the pre-stimulus baseline (−200 ms to 0 ms) was corrected. Epochs with amplitude values exceeding +60 μV at any electrode were excluded from the average. For each condition, at least 25 trials were kept for further analysis per condition. The segmented EEG for each participant was averaged for each experimental condition, resulting in ERPs which used for further statistical analyses.

Further statistical analysis was conducted in IBM SPSS Statistics 22 (IBM Corp., Armonk, NY, United States. Different sets of electrodes for FRN and P3 were chosen. The FRN was distributed in the frontal region, thus F4, Fz, F3, FC3, FCz, and FC4 were selected. P3 was distributed in the central-parietal region, thus CP3, CPz, CP4, P3, Pz, and P4 were selected. The mean amplitude of all selected electrode sites was the dependent measure. The time windows for each ERP components were chosen by visual inspection of the waveform of the grand average of all participants. Mean ERP amplitudes were determined for FRN (250–320 ms) and P3 (300–500 ms). Repeated measures ANOVA [3(Social Status: low vs. medium vs. high) × 2 (Social Mindfulness: mindful choice vs. unmindful choice)] were conducted for FRN and P3 separately. Degrees of freedom for F-ratios were corrected according to the Greenhouse-Geisser method. Statistical differences were considered significant at *p* < 0.05; *post hoc* comparisons were Bonferroni-corrected at *p* < 0.05.

## Results

### Behaviors

The mean subjective ratings of the participants’ pleasantness, how much they liked player A, and their decisions in the DG were subjected to a 3 (social status: low, medium, high) × 3 (social mindfulness: mindful, unmindful, control) repeated measures ANOVA. Descriptive data are expressed as mean ± standard error.

**FIGURE 2:**
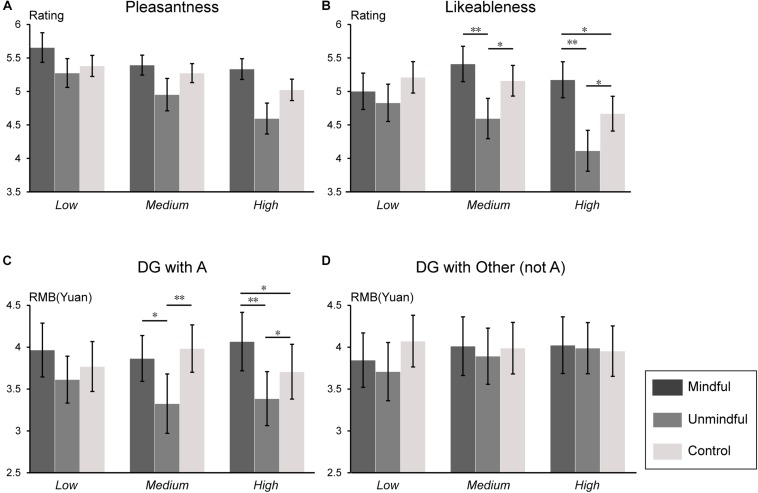
Behavioral results. Subjective ratings for each of the four questions in each condition. **(A)** Ratings for pleasantness, **(B)** ratings for likeableness of the agent A, **(C)** decision in Dictator Game with agent A, and **(D)** decisions in Dictator Game with the other (not the agent A). ^∗^*p* < 0.05 and ^∗∗^*p* < 0.01. Error bars are mean ± SE. DG refers to the Dictator Game.

For the ratings of pleasantness, the main effect of social mindfulness was significant (*F*(2,54) = 5.22, *p* = 0.008, η_*p*_^2^ = 0.16) such that participants reported the highest level of pleasantness after observing mindful choices and lowest after observing unmindful choices (mindful choices vs. unmindful choices, *p* = 0.012; mindful choices vs. control trials, *p* = 0.048; unmindful choices vs. control trials, *p* = 0.097). The main effect of social status was significant (*F*(2,54) = 4.14, *p* = 0.021, η_*p*_^2^ = 0.13) such that participants feeling more pleasant when actor A’s social status was low than when A’s status was high (Figure 2A).

For the rating of likeableness of the actor As, the main effect of social status (*F*(2,54) = 3.39 *p* = 0.045, η_*p*_^2^ = 0.10) and social mindfulness (*F*(2,54) = 5.56, *p* = 0.006, η_*p*_^2^ = 0.17) were significant. A significant interaction of social status × social mindfulness (*F*(4,108) = 3.77, *p* = 0.006, η_*p*_^2^ = 0.12) was observed. Pairwise comparison revealed that when A’s social status was medium or high, participants reported the lowest likeableness scores when the actor A made unmindful choices (Medium social status: mindful choices vs. unmindful choices, *p* = 0.009; mindful choices vs. control trials, *p* = 0.153; unmindful choices vs. control trials, *p* = 0.033. High status: mindful choices vs. unmindful choices, *p* = 0.001; mindful choices vs. control trials, *p* = 0.019; unmindful choices vs. control trials, *p* = 0.026); However, when the actor A’s social status was low, no significant effect was observed (*ps* > 0.05) (Figure 2B).

For the money allocation in DG with A, the main effect of social mindfulness was significant (*F*(2,54) = 7.518, *p* = 0.001, η_*p*_^2^ = 0.21) such that participants gave A the least money after observing A’s unmindful choice (mindful choices vs. unmindful choices, *p* = 0.009; mindful choices vs. control trials, *p* = 0.069; unmindful choices vs. control trials, *p* = 0.009). We also observed a significant interaction of social status × social mindfulness (*F*(4,108) = 0.70, *p* = 0.037, η_*p*_^2^ = 0.08). Pairwise comparison revealed that when A’s social status was medium or high, participants allocated the least money to A after observing A’s unmindful choice (Medium status: mindful choices vs. unmindful choices, *p* = 0.031; mindful choices vs. control trials, *p* = 0.238; unmindful choices vs. control trials, *p* = 0.008. High status: mindful choices vs. unmindful choices, *p* = 0.007; mindful choices vs. control trials, *p* = 0.032; unmindful choices vs. control trials, *p* = 0.026), while when A’s status was low, no significant effect was observed (*ps* > 0.05) (Figure 2C). No significant effect was observed for the money allocation in DG with the other (not A) (*ps* > 0.05) (Figure 2D).

No significant effect was observed for reaction times (*ps* > 0.05). Descriptive statistics for behavioral data were summarized in Table 1.

**TABLE 1:** Descriptive statistics for behavioral data (mean ± SE).

**Social**	**Social**	**Ratings of**	**Rating of**		**DG with the**
**Status**	**Mindfulness**	**pleasantness**	**likeableness**	**DG with A**	**other (not A)**
Low	*Mindful*	5.66 ± 0.22	5.00 ± 0.27	3.97 ± 0.32	3.85 ± 0.33
	*Unmindful*	5.27 ± 0.22	4.83 ± 0.28	3.61 ± 0.28	3.71 ± 0.35
	*Control*	5.38 ± 0.22	5.21 ± 0.23	3.77 ± 0.30	4.07 ± 0.31
Medium	*Mindful*	5.39 ± 0.16	5.41 ± 0.26	3.87 ± 0.27	4.01 ± 0.35
	*Unmindful*	4.95 ± 0.24	4.60 ± 0.30	3.33 ± 0.36	3.89 ± 0.34
	*Control*	5.27 ± 0.14	5.16 ± 0.23	3.98 ± 0.28	3.99 ± 0.31
High	*Mindful*	5.33 ± 0.16	5.17 ± 0.27	4.07 ± 0.35	4.02 ± 0.34
	*Unmindful*	4.60 ± 0.23	4.11 ± 0.31	3.39 ± 0.32	3.99 ± 0.31
	*Control*	5.02 ± 0.16	4.67 ± 0.27	3.71 ± 0.33	3.95 ± 0.30

### ERPs

Although we included the control trials in the statistical analysis of the behavioral ratings, we did not include them in the ERP analysis. The reason is as follows: Participants may perceive the actor’s personal preference in the control trials (e.g., the actor likes the color white over the color blue) instead of whether the actor’s choice is socially mindful. Theoretically, at the behavioral level, any perceived personal preference in these kinds of trivial decisions would not dramatically influence the subjective ratings and thus can be a nice baseline. However, at the neural level, if the actor’s choice violates the observer’s prediction about the actor’s personal preference, it could also modulate FRN and/or P3. As this is essentially different from the effect induced by social mindfulness, we removed these trials from the ERP analysis.

#### FRN (250–320 ms)

**FIGURE 3:**
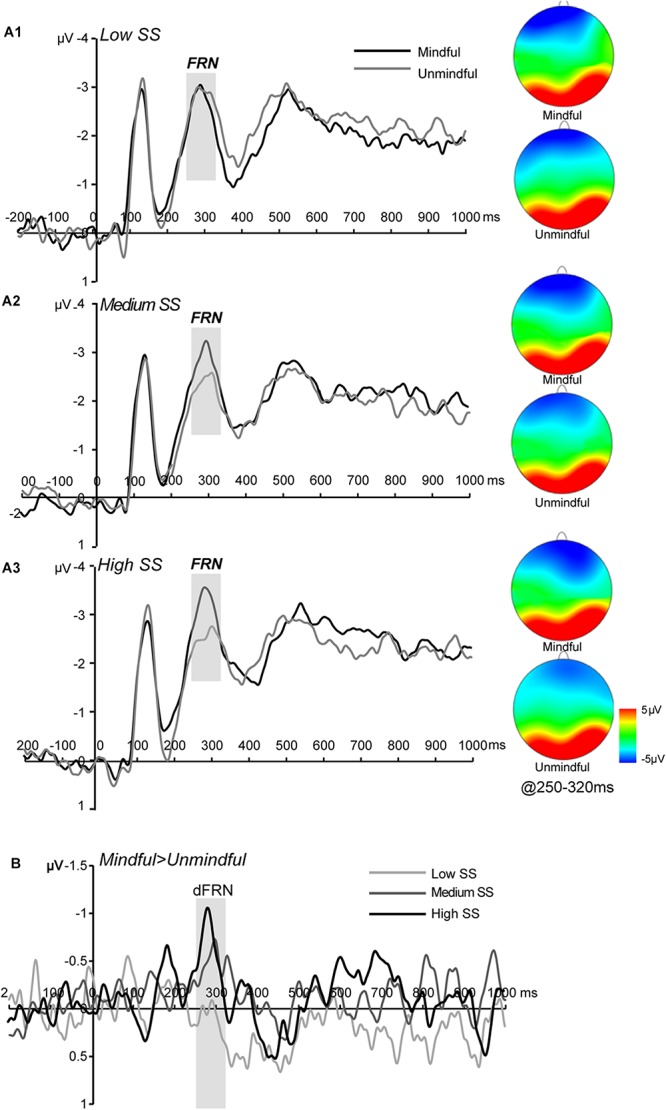
ERP results for FRN **(A1–A3)** Grand averaged FRN at frontocentral regions (averaged F3, Fz, F4, FC3, FCz, and FC4) for low, medium, and high social status (SS) under mindful and unmindful conditions and the corresponding topography. **(B)** The dFRN (mindful- unmindful) for each SS level.

The main effect of social mindfulness was significant (*F*(1,27) = 5.45, *p* = 0.027, η_*p*_^2^ = 0.16) such that FRN amplitudes were significantly more negative in response to mindful choices than to unmindful choices. Critically, we observed a significant social status × social mindfulness interaction (*F*(2,54) = 3.53, *p* = 0.038, η_*p*_^2^ = 0.11). Pairwise comparison revealed that when actor A’s status was medium or high, FRN was larger for mindful choices than for unmindful choices (medium status: *p* = 0.033; high status: *p* = 0.013). However, when A’s status was low, FRN amplitude did not significantly differ between mindful and unmindful choices (*p* = 0.719) (Figures 3A,B).

#### P300 (300–500 ms)

The main effect of social mindfulness was not significant (*F*(1,27) = 0.002, *p* = 0.963, η_*p*_^2^ = 0.001). The main effect of social status was significant (*F*(2,54) = 3.91, *p* = 0.030, η_*p*_^2^ = 0.12) such that high status condition elicited larger P3 amplitudes than medium status condition. The social status × social mindfulness interaction was not significant (*F*(2,54) = 0.608, *p* = 0.531, η_*p*_^2^ = 0.02).

Descriptive statistics for ERP data were summarized in Table 2.

**TABLE 2:** Descriptive statistics for ERP data (mean ± s.e.).

**Social**	**Social**		
**Status**	**Mindfulness**	**FRN (μV)**	**P3 (μV)**
Low	*Mindful*	−2.71 ± 0.45	3.86 ± 0.49
	*Unmindful*	−2.79 ± 0.47	4.12 ± 0.64
Medium	*Mindful*	−3.03 ± 0.50	4.15 ± 0.63
	*Unmindful*	−2.56 ± 0.48	4.00 ± 0.68
High	*Mindful*	−3.24 ± 0.46	4.56 ± 0.61
	*Unmindful*	−2.56 ± 0.45	4.42 ± 0.71

#### The Self-SoMi Index Correlated With Subjective Ratings and FRN Amplitudes

For the participant’s own SoMi index, we firstly run a one-sample *t*-test between participant’s SoMi index and the baseline 0.5 to examine whether the participants showed social mindfulness on the group level. Results showed that their SoMi index was significantly larger than 0.5 (0.59 ± 0.04, *t* (26) = 2.30, *p* = 0.029).

To explore how the participants’ own SoMi index may influence they feel about other’s social mindfulness, we run Spearman correlations analysis between the participant’s own SoMi scores and their ratings of pleasantness, likeableness of the actor A and decisions in the DG. To investigate how one’s own socially mindful character is related to the ERP components elicited by observing other’s social mindfulness, we performed Spearman’s correlation analyses between the FRN amplitudes and the participants’ self SoMi indices, separately for each condition. No significant correlation survived correction for multiple comparisons was observed.

## Discussion

This study investigated two critical issues with regard to the perception of social mindfulness. First, we compared the behavioral and neural responses triggered by observing others’ socially mindful/unmindful choices. Results suggested a small gesture of social mindfulness (i.e., making choices that preserve another’s sense of control) can be detected and appreciated. At the behavioral level, after observing socially mindful choices, observers felt better, rated the actors as more likable, and behaved more cooperatively. At the neural (ERP) level, socially mindful choices elicited more negative FRN than socially unmindful choices did. Second, we evaluated the effect of social status on the perception and evaluation of social mindfulness. Results showed that as the actor’s social status increased, the social mindfulness becomes more salient.

Results from the subjective ratings are consistent with previous findings (Van Doesum et al., 2013; Van Lange and Van Doesum, 2015; Dou et al., 2018a, b). Interestingly, we found that the effect of social mindfulness on subsequent cooperative decision-making (i.e., allocating money in the DG) was only significant when the co-player was the actor. When the co-player was someone else, the previously observed social mindfulness did not significantly affect the observer’s decisions. These results imply that the effect of social mindfulness on interpersonal decision-making was not generalized to others.

FRN has been suggested to be sensitive to outcome signals and is usually enhanced when stimuli are negative/unfavorable than when they are positive/favorable (Holroyd and Coles, 2002; Liu and Gehring, 2009). Although the present results indicate that FRN amplitude does differentiate socially mindful from socially unmindful choices in certain contexts, the findings contradict the idea that FRN is enhanced by negative stimuli. Indeed, we found that the positive and favorable mindful choices elicited significantly larger FRN than did the negative and unfavorable unmindful choices. There are two possible explanations. The first is that socially mindful choices are more unexpected and uncertain than unmindful choices. FRN amplitude enhancement is often interpreted as a saliency prediction error that reflects the discrepancy between observed outcomes and an observer’s predictions (Alexander and Brown, 2011; Pfabigan et al., 2011; Talmi et al., 2013; Hauser et al., 2014). Previous studies found when participants were alone or the last to choose (i.e., without any social pressure), most preferred the unique option, meaning that the default choice is to pick the unique item (Yamagishi et al., 2008; Hashimoto et al., 2011). The preference for uniqueness has been demonstrated in both western and eastern cultures, and observers should thus tend to predict that others would also choose a unique item if given the chance. A socially mindful choice (i.e., not choosing the unique item) could be unexpected, which would explain the larger FRN that we observed for mindful choices. Besides, for the observers, the mindful choices are always more uncertain than unmindful choices. In the mindful choices, participants are left with multiple choices while in the unmindful choices, participants are left with one unique choice. The uncertainty of mindful choice would also contribute to the enhanced FRM. The second explanation is that socially mindful choice is more motivationally salient than unmindful choices. Unless an actor consistently behaves unmindfully, observers are reluctant in judging others as hostile and uncooperative in the SoMi context (Van Lange and Van Doesum, 2015). As proposed by Van Lange and Van Doesum (2015), social life is strongly colored by noise, and using a benefit-of-the-doubt-approach is more reasonable and less arbitrary when judging others through implicit clues (such as social mindfulness), especially when judging others as negative. The socially unmindful choice is, therefore, less motivational than mindful choices, which could explain the smaller FRN that we observed. However, it should be noticed that whether this “positive bias” exists in the processing of social mindfulness needs further confirmation with larger sample size and specified design. For now, this bias can only be implied from previous findings.

For the P3 component, no significant effect or interaction of social mindfulness was observed, which indicates that the perception of others’ social mindfulness is fast and innate. The neural responses triggered by observing other’s mindful/unmindful behaviors mostly happen during the early, rapid, and automatic processing stage, but not the later, cognitive evaluation stage (Wu and Zhou, 2009; Zhou et al., 2010). Thus, social mindfulness can be rapidly detected and processed, as reflected by FRN, although it does not seem to receive later, more elaborate evaluation. There might be two reasons. First, compared with the explicit, non-cooperative behaviors in the traditional cooperation games (e.g., reject in the UG), social mindful/unmindful behaviors are more subtle. Second, the social mindfulness/hostility indicated in the SoMi task would only become explicit and important when they appear repetitively and consistently. However, in the present study, participants were informed that the actor in each trial was a different individual. This might reduce the participants’ motivation to cognitively evaluate the observed behavior.

With respect to social status, we found that at both behavioral and neural levels, the effect of social mindfulness was significant when the actor’s social status was medium or high, but not when the actor has low-status. This might because more attention was allocated to process information about others with higher social status. Previous studies reported that individuals with low social status showed more concern about the feelings and emotions of others, as well as more prosocial behaviors compared with those who had high social status (Guinote, 2001; Guinote et al., 2015). Thus when higher status individuals behaving socially mindful, it might be more unexpected and thus be more salient. The observed significant interactions indicated that the higher status the actor is, the more sensitive our brains are to their social mindfulness.

In sum, the present study explored how social mindfulness was perceived and evaluated at both behavioral and neural levels. These results showed that social mindful/unmindful choices can significantly influence an observer’s mood, how much they like the people, and even future social decisions they might make involving them. Social mindfulness can be rapidly detected and differentiated in early, automatic processing, as reflected in the FRN. However, it does not seem to be further evaluated at more elaborate, cognitive processing stages reflected in P3. We also found that sensitivity to others’ social mindfulness was strongly modulated by social status such that observers were most sensitive to the behavior of high-status individuals. These findings suggested that low-cost cooperative behaviors such as social mindfulness can be detected and appreciated by our brain, which may result in better mood and more cooperative behaviors in the perceivers.

## Data Availability Statement

The datasets generated for this study are available on request to the corresponding authors.

## Ethics Statement

The studies involving human participants were reviewed and approved by the Medical Ethical Committee of the Medical School in Shenzhen University. The patients/participants provided their written informed consent to participate in this study.

## Author Contributions

FC and JL designed the research. JL and XH collected the data. JL, CL, and XH analyzed the data. FC, QG, JL, and X-RQ wrote the manuscript.

## Conflict of Interest

The authors declare that the research was conducted in the absence of any commercial or financial relationships that could be construed as a potential conflict of interest.
